# Supplementary material for: Creating resistance to the whitefly Bemisia tabaci in cassava through RNAi-mediated targeting of multiple insect metabolic processes
Source: Front Plant Sci. 2026 May 22;17:1822258. doi: 10.3389/fpls.2026.1822258 (PMC13236953; doi:10.3389/fpls.2026.1822258)
Supplement: Supplementary Figure 2 — Transverse sections of stem and storage roots indicating GUS localization, particularly in phloem tissues. [file Presentation2.pptx]

## Slide 1
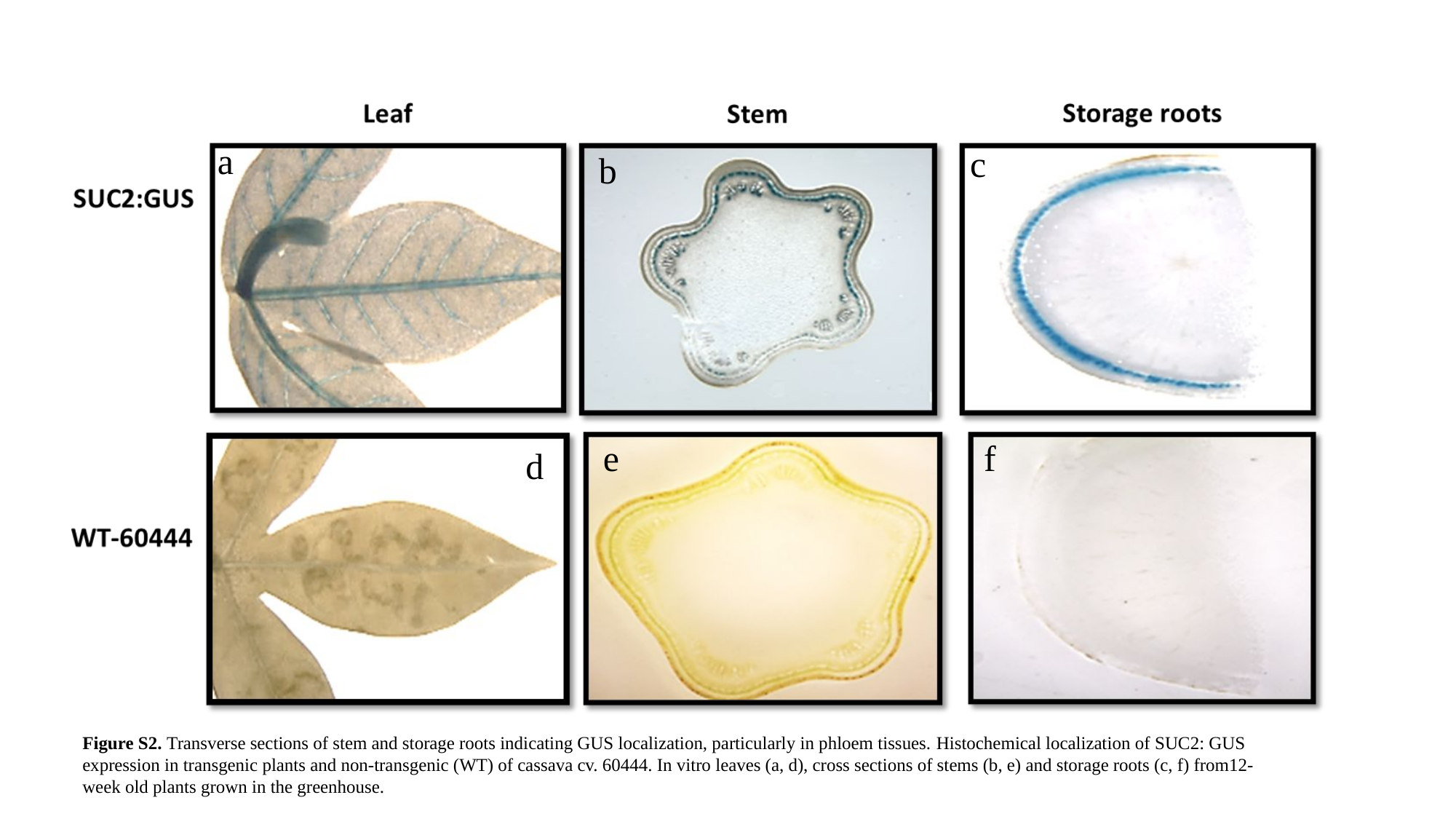

a
c
b
f
e
d
Figure S2. Transverse sections of stem and storage roots indicating GUS localization, particularly in phloem tissues. Histochemical localization of SUC2: GUS expression in transgenic plants and non-transgenic (WT) of cassava cv. 60444. In vitro leaves (a, d), cross sections of stems (b, e) and storage roots (c, f) from12-week old plants grown in the greenhouse.
