# Supplementary material for: Creating resistance to the whitefly Bemisia tabaci in cassava through RNAi-mediated targeting of multiple insect metabolic processes
Source: Front Plant Sci. 2026 May 22;17:1822258. doi: 10.3389/fpls.2026.1822258 (PMC13236953; doi:10.3389/fpls.2026.1822258)
Supplement: Supplementary file 6 [file Table4.docx]

**Table S4.** *P*-values of comparing the total number of developing nymphs between independent transgenic RNAi events and a control event expressing dsRNA against the GFP gene. Nonparametric multiple comparisons of treatment groups versus the control were conducted using Steel’s method. Results are reported as significant (yellow and green background) when the adjusted *P* value was < 0.05. Yellow background = lower total nymphs than control; Green background = higher total nymphs than control.

| **Trangenic event** | ***P*-Value** | **Trangenic event** | ***P*-Value** | **Trangenic event** | ***P*-Value** |
| --- | --- | --- | --- | --- | --- |
| DWF11-N13001 | 0.06702964 | DWF59-N13005 | 0.050431729 | DWF66-N13005 | 0.999981389 |
| DWF11-N13004 | 0.999998982 | DWF60-N13001 | 1 | DWF66-N13006 | 0.11461249 |
| DWF11-N13008 | 0.322286257 | DWF60-N13002 | 0.082549259 | DWF66-N13007 | 0.999474473 |
| DWF15-N06001 | 1 | DWF60-N13003 | 0.999998249 | DWF66-N13008 | 0.113270494 |
| DWF15-N06002 | 0.116824461 | DWF60-N13004 | 0.02993322 | DWF66-N13009 | 0.999804573 |
| DWF15-N06003 | 1 | DWF60-N13006 | 0.499774867 | DWF66-N13013 | 0.041563402 |
| DWF15-N13001 | 1 | DWF60-N13007 | 0.997193801 | DWF66-N13014 | 0.057176284 |
| DWF15-N13002 | 0.854635083 | DWF60-N13009 | 1 | DWF66-N13015 | 0.021288519 |
| DWF55-N13003 | 0.964368873 | DWF61-N13001 | 0.90172867 | DWF67-N13001 | 0.308083402 |
| DWF55-N13004 | 0.985119768 | DWF61-N13002 | 0.827188485 | DWF67-N13002 | 0.986983552 |
| DWF55-N13005 | 0.044381182 | DWF61-N13003 | 0.938246511 | DWF67-N13003 | 0.123645722 |
| DWF55-N13009 | 0.999618424 | DWF61-N13005 | 0.011275423 | DWF67-N13004 | 0.057176284 |
| DWF55-N13011 | 1 | DWF61-N13007 | 0.077712518 | DWF67-N13005 | 0.114557713 |
| DWF55-N13012 | 1 | DWF61-N13008 | 1 | DWF67-N13006 | 0.308262727 |
| DWF55-N13013 | 0.571712272 | DWF61-N13010 | 0.999999468 | DWF67-N13009 | 0.01302201 |
| DWF56-N13001 | 0.999999999 | DWF61-N13011 | 0.626261667 | DWF67-N13011 | 0.517656796 |
| DWF56-N13002 | 0.766046621 | DWF62-N13001 | 0.01121154 | DWF67-N13010 | 0.571606064 |
| DWF56-N13003 | 0.999999999 | DWF62-N13003 | 0.022829545 | DWF68-N13004 | 0.026162466 |
| DWF56-N13004 | 0.697905319 | DWF62-N13004 | 0.035549817 | DWF68-N13005 | 0.093517822 |
| DWF56-N13005 | 0.99781266 | DWF62-N13005 | 1 | DWF68-N13006 | 0.999988028 |
| DWF56-N13006 | 0.268690026 | DWF62-N13008 | 0.011275423 | DWF68-N13007 | 0.013030066 |
| DWF57-N13001 | 0.986994443 | DWF63-N13001 | 0.130844659 | DWF68-N13008 | 1 |
| DWF57-N13002 | 0.180440935 | DWF63-N13002 | 1 | DWF68-N13009 | 0.999999813 |
| DWF57-N13003 | 0.644507072 | DWF63-N13003 | 0.464517657 | DWF68-N13011 | 0.945605224 |
| DWF57-N13004 | 0.765082047 | DWF63-N13004 | 0.553233892 |  |  |
| DWF57-N13005 | 1 | DWF63-N13005 | 1 |  |  |
| DWF57-N13006 | 0.732784871 | DWF63-N13009 | 0.145988344 |  |  |
| DWF57-N13007 | 0.011275423 | DWF64-N13001 | 0.958750137 |  |  |
| DWF57-N13008 | 0.011275423 | DWF64-N13002 | 0.992226484 |  |  |
| DWF57-N13009 | 0.952588084 | DWF65-N13003 | 0.073122488 |  |  |
| DWF58-N13001 | 0.999999972 | DWF65-N13004 | 0.011268312 |  |  |
| DWF58-N13002 | 1 | DWF65-N13005 | 0.055352205 |  |  |
| DWF58-N13003 | 0.94574187 | DWF65-N13006 | 0.341909771 |  |  |
| DWF58-N13004 | 0.765482011 | DWF66-N13001 | 0.281162708 |  |  |
| DWF59-N13001 | 0.999999899 | DWF66-N13002 | 0.921427357 |  |  |
| DWF59-N13003 | 1 | DWF66-N13003 | 0.172543744 |  |  |
